# The pivotal role of cholesterol absorption inhibitors in the management of dyslipidemia

**DOI:** 10.1186/1476-511X-3-22

**Published:** 2004-10-07

**Authors:** Moutasim H Al-Shaer, Nabil E Choueiri, Ehab S Suleiman

**Affiliations:** 1 Department of Internal Medicine, and Human Cardiovascular Physiology Laboratory University of Iowa College of Medicine Iowa City, Iowa 52242-1009, USA

**Keywords:** Ezetimibe, Zetia, Cholesterol Absorption Inhibitors, Dyslipidemia, Hyperlipidemia, Hypercholesterolemia, Atherosclerosis, Sitosterolemia, Pleiotropic Effects of Statins, homozygous familial hypercholesterolemia

## Abstract

Elevated low-density lipoprotein (LDL)-cholesterol is associated with a significantly increased risk of coronary heart disease. Ezetimibe is the first member of a new class of selective cholesterol absorption inhibitors. It impairs the intestinal reabsorption of both dietary and hepatically excreted biliary cholesterol. Ezetimibe is an effective and safe agent for lowering LDL-C and non HDL-C. Short term clinical trials have established the role of ezetimibe monotherapy and its use in combination with statins. Furthermore, ezetimibe and statin combination therapy increased the percentage of patients who achieved their LDL-C treatment goal.

Studies using surrogate markers of atherosclerosis have suggested a possible role of ezetimibe in combating atherosclerosis. Ezetimibe provides an effective therapeutic strategy for the management of homozygous familial hypercholesterolemia (HoFH) and sitosterolemia. The lack of outcomes and long term safety data is attributed to the relatively recent introduction of this medication.

## Background

Over 60 million Americans suffer from cardiovascular disease (CHD). The incidence of CHD and stroke has been on the rise partly because of the increase in life expectancy and the explosive epidemic of diabetes and the metabolic syndrome [1]. CHD is responsible for about 38% of the overall mortality in the United States making it the number one killer of Americans [2].

Animal and human studies have established the role of cholesterol in the development and progression of atherosclerosis. LDL-cholesterol (LDL-C) constitutes approximately 60–70 % of total serum cholesterol. Epidemiological studies directly implicated LDL-C to the development of atherosclerosis and CHD. Furthermore, LDL-C level appears to be directly related to the development and recurrence of CHD [3].

Animal studies suggested a protective effect of low LDL-C against atherosclerosis [2]. Multiple human trials examining the relationship of LDL-C lowering in primary and secondary prevention of CHD have demonstrated the impact of reducing LDL-C levels on decreasing CHD and CHD related mortality [4-8].

Most of the landmark CHD prevention trials involved the use of statin medications. LDL-C remains the primary target of treatment in most instances, and statins are the mainstay of LDL-C lowering treatment [9].

The National Cholesterol Education Program/ Adult Treatment Panel III (NCEP/ATP III) updated guidelines (table 1) for detection and treatment of dyslipidemia envisioned LDL-C below 100 mg/dL to be optimal for all patient risk categories. These more aggressive guidelines resulted in doubling of the number of patients that are not at target LDL-C levels as compared to previous guidelines [2]. Recent NCEP/ATP III update data suggested even lower LDL-C levels than previously advocated, making it harder to achieve the treatment in many instances and recommended the use of combination therapy if needed to help achieve the treatment targets. The NCEP/ATP III update emphasized "the lower, the better" hypothesis [10].

**Table 1:** Synopsis of the updated ATP III LDL-C Goals and Cut-points for TLC and Drug Therapy in Different Risk Categories and Proposed Modifications Based on Recent Clinical Trial Evidence

Risk Category	Goal	TLC	Drug Therapy
*High risk*: CHD or CHD risk equivalents (10-year risk >20%)	< 100 mg/dL (optional goal: <70 mg/dL)	≥ 100 mg/dL	≥ 100 (<100 mg/dL: consider drug options)
*Moderately high risk*: 2+ risk factors (10-year risk 10% to 20%)	< 130 mg/dL	≥ 130 mg/dL	≥ 130 mg/dL(100–129 mg/dL; consider drug options)
*Moderate risk*: 2+ risk factors (10-year risk <10%)	< 130 mg/dL	≥ 130 mg/dL	≥ 160 mg/dL
*Lower risk*: 0–1 risk factor	< 160 mg/dL	≥ 160 mg/dL	≥ 190 mg/dL (160–189 mg/dL: LDL-lowering drug optional)

## Cholesterol Absorption inhibitors

Ezetimibe, a cholesterol absorption inhibitor, is the first agent of a new class of lipid-lowering compounds that selectively inhibits the intestinal absorption of cholesterol and related phytosterols. Ezetimibe undergoes extensive glucuronidation to an active metabolite in the intestinal mucosa [11]. Ezetimibe acts on brush border of the small intestine and decreases biliary and dietary cholesterol from the small intestine uptake into the enterocytes. Ezetimibe is primarily metabolized in the small intestine and liver via glucuronide conjugation with subsequent biliary and renal excretion [12]. Ezetimibe does not affect the absorption of fat-soluble vitamins, triglycerides, or bile acids [13].

After oral administration, ezetimibe is absorbed and extensively conjugated to a pharmacologically active phenolic glucuronide (ezetimibe-glucuronide) [14], the drug and its metabolite have a half-life of approximately 22 hours [8]. Concomitant food administration (high fat or non-fat meals) had no effect on the extent of absorption of ezetimibe when administered in the 10-mg clinical dose [15]. Ezetimibe and ezetimibe-glucuronide are highly bound (>90%) to human plasma proteins [16].

Plasma concentrations for total ezetimibe were about 2-fold higher in older individuals (>65 years), levels were similar in adolescents to healthy adults and may be higher in women than in men [8]. In patients with severe renal disease, ezetimibe level was increased approximately 1.5-fold, compared to healthy controls [17]. Ezetimibe had no significant effects on the bioavailability of warfarin, fenofibrate, HMG CoA reductase inhibitors, or digoxin [16,18-20].

Adverse experiences were reported in approximately 2% of patients treated with ezetimibe and included fatigue, arthralgia, diarrhea, abdominal pain and back pain. Angioedema and rash were reported after general clinical use of this medication [16]. With co-administration of ezetimibe and statins the adverse event profile was similar to that for statins alone. In a recently published case report, the authors described two patients whose creatinine kinase (CK) increased after the addition of ezetimibe to statin therapy causing one of the patients to experience myalgia and tendinopathy. This finding raises the question of whether ezetimibe can be implicated in precipitating increased risk of stain-associated myopathy [21].

## The Role of Ezetimibe in Clinical Practice

### Indications for use

1. Primary Hypercholesterolemia (heterozygous familial and non-familial), Ezetimibe is indicated in this case for use as both mono and combination therapy.

2. The reduction of elevated total-C and LDL-C levels in patients with homozygous familial hypercholesterolemia (HoFH) either as primary or as an adjunct to other lipid-lowering treatments.

3. In patients suffering from Homozygous Sitosterolemia, as adjunctive therapy to diet for the reduction of elevated sitosterol and campesterol levels in patients with homozygous familial sitosterolemia.

## Monotherapy

Multiple studies conducted to examine the effects of ezetimibe monotherapy have concluded that this drug was effective in lowering LDL-C versus placebo.

Analysis of multicenter, double-blind, placebo-controlled trials demonstrated that ezetimibe at the 10 mg once daily clinically approved dose significantly modified cholesterol and cholesterol subtypes in patients with hypercholesterolemia when compared to placebo. Ezetimibe significantly lowered total-Cholesterol (TC) (12 %), LDL-C (18 %), apolipoprotein B (Apo B) (15 %), and triglycerides (TG) (7%) and increased high density lipoprotein (HDL-C) (3.5%) [22-24]. Lipoprotein (a) [Lp (a)] was not significantly affected by Ezetimibe 10 mg once a day treatment [25].

In a case series report, we analyzed the effects of Ezetimibe on cholesterol particle size and number using NMR technology (Lipo science, Raleigh, NC). We found that Ezetimibe lowered cholesterol particle number by a mean 26 % and had no significant effect on cholesterol particle size [26].

The effects of ezetimibe on cholesterol and its subtypes were not influenced by risk-factor status, gender, age, race, time of administration, or baseline lipid profile [22]. The overall incidence of adverse effects with ezetimibe monotherapy was similar to placebo.

## Combination therapy

The struggle to achieve the NCEP/ATP III guidelines LDL-C goals through primary utilization of statins is often frustrating for the clinician. In a study to examine the efficacy of statin titration on attainment of LDL-C goal, the authors concluded that for high risk patients, approximately half were able to achieve their LDL-C goal at the appropriate statin starting dose, and only one third of the titration group were able to achieve the NCEP/ATP III cholesterol goal [27]. Now with the very recent publication of the update to the NCEP/ATP III, clinicians are faced with even lower goals of LDL-C, Making combination therapy a must in more cases than previously advocated by the NCEP/ATP III [10].

HMG-CoA reductase inhibitors (statins) act on the rate-limiting step to inhibit HMG-CoA conversion to mevalonate, effectively decreasing LDL-C synthesis. They result in a decrease of LDL-C ranging between 30–60 %, depending on the individual statin and the dose administered. Statin induced LDL-C lowering appears to be effective in reducing CHD and CHD related mortality and morbidity. The extent of CHD and CHD related events reduction is proportionate to the extent of LDL-C reduction [10,28]. LDL-C reduction trials have demonstrated a reduction in CHD related events by approximately 20–40% [4-8].

Reasons to initiate combination therapy to treat hyperlipidemia include: further LDL-C lowering, reducing side effects related to higher doses of statins, modifying other risk factors besides LDL-C such as HDL-C and TG.

Increasing the dose of statins has a limited effect on reducing LDL-C, as it is well established that doubling the dose of a statins leads to a 5 % more reduction in total TC and 7 % more reduction in LDL-C with each doubling [29].

Although statins have demonstrated similarity in CHD related events, they are heterogeneous not only in LDL-C lowering efficacy but also in their safety profiles. The bulk of the statins effect on LDL-C occurs at the initial recommended dose and they are safer when used at doses below the maximal recommended dose.

Statins are the most effective drugs known to modify LDL-C, but in terms of HDL-C and TG modifying capacity, other classes of lipid lowering medications used alone or in combination with statins offer higher efficacy.

The metabolism of cholesterol is an intricate process that involves both produced and ingested cholesterol. The mechanism of action of HMG-CoA reductase inhibitors affects the production of cholesterol, whereas that of cholesterol absorption inhibitors affects absorbed cholesterol, thereby offering potential synergism of action when the medication are used in combination. Trials examining the efficacy have demonstrated synergism and consistency in LDL-C lowering in the absence pharmacokinetic interaction between the statins and ezetimibe.

In a relatively large, multicenter study, involving patients with primary hypercholesterolemia already receiving statin monotherapy (but who had not met their NCEP ATP II target LDL-C goal), patients were randomized to receive either ezetimibe or placebo in addition to their current statin therapy. At the conclusion of this 8 week study, the ezetimibe and statin groups were found to have a significantly lower total-C, LDL-C, Apo B, and TG, and increased HDL-C when compared to the statin only and placebo groups. Furthermore, LDL-C reductions induced by ezetimibe were generally consistent across all statins groups [30].

Another multicenter, double-blind, randomized trial examined the effects of ezetimibe on patients suffering from (HoFH). At the initiation of the trial patients were receiving either atorvastatin or simvastatin. The addition of ezetimibe reduced LDL-C by an additional 20.5 % in contrast to only 6.7 % reduction that resulted from doubling the statin dose [31]. Similar results were demonstrated in high-risk patients with familial heterozygous hypercholesterolemia (HeFH) [32].

The addition of ezetimibe to statins is superior to treatment with statins alone in lowering non-HDL-C, ezetimibe co-administered with simvastatin lowered non-HDL-C by 47.1% whereas, simvastatin monotherapy lowered non-HDL-C by 33.6% when results were pooled across different doses [33].

In terms of modifying risk factors other than LDL-C, the co-administration of ezetimibe with statins had a more favorable effect on HDL-C and TG when compared to statins therapy alone [33]. In conclusion, the addition of ezetimibe to statins produced further lowering of LDL-C of approximately 15–20 % with no apparent increase in side effects. This effect was superior to that observed by doubling the dose of the statins. Furthermore, the lowering produced was consistent.

Ezetimibe co-administration with fibric acid derivatives was examined in a randomized, evaluator-blind, placebo-controlled, parallel-group study of 32 healthy hypereholesterolemics. Ezetimibe co-administration with fenofibrate was found to produce clinically significant reductions in LDL-C (36.3%) compared to the fenofibrate group (22.3%) with a more favorable TG and HDL-C profile [34].

## Sitosterolemia

Sitosterolemia is a rare inherited disorder caused by mutation in either the ABCG5 or ABCG8 genes located on chromosome (2p21) [35]. First described by Bhattacharyya and Connor in 1974 in two sisters of German and German-Swiss ancestry with normal mental development. The patients presented with tendinous and tuberous xanthoma and elevation of beta-sitosterol, campesterol and stigmasterol (plant sterols) in the blood [36]. Affected individuals have increased intestinal absorption of plant sterols (mainly sitosterol) that are usually absorbed in minute amounts in normal individuals. Additionally, these patients have diminished clearance of plant sterols, leading to very high levels of plant sterols in the plasma. Patients suffering from sitosterolemia have severely depressed hepatic cholesterol biosynthesis, and decreased levels of HMG-CoA reductase enzyme [37].

Clinical manifestations include: tendon and tuberous xanthomas, episodes of hemolysis, accelerated atherosclerosis, and premature coronary artery disease. It is important to note that close to 50 % of these patients have normal cholesterol levels [38-41].

A recently reported trial demonstrated that treatment with ezetimibe reduces plant sterol levels in patients with sitosterolemia. The authors reported a decrease in sitosterol concentrations by 21% and campesterol by 24 % [42].

## Ezetimibe and atherosclerosis

It is generally accepted that atherosclerosis is an inflammatory disorder. It is believed that the atherosclerotic process begins with endothelial cell activation, which is triggered by multiple factors such as oxidized lipoproteins. Cholesterol lowering agents as a group have demonstrated great efficacy in prevention and cessation of the progression of atherosclerosis. The efficacy of ezetimibe in monotherapy or in combination on CHD morbidity and mortality has not been well established.

One of the unique features of cholesterol absorption inhibitors is their ability to modify post-prandial hyperlipidemia. There is increasing evidence that post-prandial lipoproteins (particularly cholesterol-rich chylomicron remnant) are atherogenic. Ezetimibe has the potential to reduce the cholesterol content of chylomicrons by up to 60% [44], which may lead to a lower atherogenic potential of chylomicron remnants [43].

High-sensitivity C-reactive protein (hs-CRP) is an inflammatory mediator whose levels correlate with increased coronary risk. Ezetimibe co-administered with simvastatin resulted in significant incremental decreases in hs-CRP in patients with primary hypercholesterolemia. Changes in individual lipid parameters did not explain the observed decreases in hs-CRP and were possibly consistent with an additional anti-inflammatory effect compared with simvastatin monotherapy [45].

In a prospective trial to study effects of ezetimibe co-administered with atorvastatin in patients with primary hypercholesterolemia, ezetimibe plus atorvastatin significantly provided an additional (10%) lowering of hs-CRP versus atorvastatin alone [46].

## The unanswered questions

Ezetimibe and its class of cholesterol absorption inhibitors are new, and there is a lack of outcomes data to explore whether its cholesterol modifying effects will translate to lower CHD mortality and morbidity. The safety of this medication has not yet been established with long term trials data as most of the studies conducted were short term. With the advent and increased utilization of combination therapy in the management of dyslipidemia, further trials are needed to explore the efficacy, indications and safety profile of ezetimibe use in combination with Peroxisome proliferator-activated receptors (PPARs), niacin and bile acid resins.

The increased popularity of special weight loss diets such as the high protein diet, poses questions of whether such diets will alter the efficacy or safety of cholesterol absorption inhibitors.

Finally, the efficacy of statins in reducing CHD related events has lead to the controversial hypothesis regarding whether or not statins poses a pleiotropic (non lipoprotein) effect. If a pleiotropic effect exists, one might argue that a statin at a higher dose might be more beneficial than combination therapy producing the same effect.

## Conclusions

Ezetimibe is the first clinically approved cholesterol absorption inhibitor. It is effective in lowering LDL-C as monotherapy or in combination with statins. The use of combination LDL-C lowering medication is expected to become a much more common modality of treatment, especially after the recent NCEP/ATP III update. Ezetimibe offers further lowering of LDL-C and non HDL-C that is consistent and probably safer than increasing the dose of the individual statin. It also provides another effective treatment option for HoFH and sitosterolemia patients. Because of its recent introduction, we still lack both outcomes and long term safety data.
